# ﻿Three novel species of *Distoseptispora* (Distoseptisporaceae) isolated from bamboo in Jiangxi Province, China

**DOI:** 10.3897/mycokeys.88.79346

**Published:** 2022-03-22

**Authors:** Zhi-Jun Zhai, Jun-Qing Yan, Wei-Wu Li, Yang Gao, Hai-Jing Hu, Jian-Ping Zhou, Hai-Yan Song, Dian-Ming Hu

**Affiliations:** 1 Bioengineering and Technological Research Centre for Edible and Medicinal Fungi, College of Bioscience and Bioengineering, Jiangxi Agricultural University, Nanchang, Jiangxi 330045, China Jiangxi Agricultural University Nanchang China; 2 Jiangxi Key Laboratory for Conservation and Utilization of Fungal Resources, College of Bioscience and Bioengineering, Jiangxi Agricultural University, Nanchang, Jiangxi 330045, China Jiangxi Agricultural University Nanchang China; 3 Key Laboratory of Crop Physiology, Ecology and Genetic Breeding, Ministry of Education of the P. R. China, Jiangxi Agricultural University, Nanchang, Jiangxi 330045, China Jiangxi Agricultural University Nanchang China

**Keywords:** Hyphomycetes, phylogenetic analysis, *Sordariomycetes*, taxonomy, three new taxa

## Abstract

Decaying bamboo in freshwater is a unique eco-environment for fungi. Three new Distoseptispora (Distoseptisporaceae) species, *D.meilingensis*, *D.yongxiuensis* and *D.yunjushanensis* from submerged decaying bamboo culms in Jiangxi Province, China, were discovered, based on phylogenetic analyses and morphological characters. The combined data of ITS-LSU-SSU-*Tef*1 sequences were used to infer the phylogenetic relationship between *D.meilingensis*, *D.yongxiuensis*, *D.yunjushanensis* and related species. Both molecular analyses and morphological data supported *D.meilingensis*, *D.yongxiuensis* and *D.yunjushanensis* as three independent taxa.

## ﻿Introduction

*Distoseptispora* was established by Su et al. (2016) as the single genus in *Distoseptisporaceae*. This genus morphologically resembles *Ellisembia* and *Sporidesmium* (Subramanian 1992; Shenoy et al. 2006; Yang et al. 2018), while they are not in sister clades in molecular phylogenetic trees (Su et al. 2016; Luo et al. 2019; Hyde et al. 2020, 2021). Multigene analysis showed that *Distoseptispora* formed a stable and well-supported clade within *Distoseptisporales* as a sister clade to *Aquapteridospora* (Luo et al. 2019; Hyde et al. 2020, 2021). *Aquapteridospora* has been raised as a new family *Aquapteridosporaceae* for the divergence time (110 million years ago (mya)) falling within the family-level range (50–130 mya) (Hyde et al. 2021). *Aquapteridospora* and *Distoseptispora* are similar in having macronematous, mononematous, unbranched conidiophores, mono- or polyblastic, holoblastic, conidiogenous cells and acrogenous, solitary conidia. *Distoseptispora* can easily be distinguished from *Aquapteridospora* by its short conidiophores and obclavate or cylindrical, rostrate, euseptate or distoseptate conidia. Additionally, *Distoseptispora* has terminal conidiogenous cells which lack circular scars (Hyde et al. 2021).

*Distoseptispora* was regarded as saprobic lignicolous fungal genus, which has the ability to decompose lignocelluloses in wood (Wong et al. 1998; Hyde et al. 2016). In recent years, the number of new taxa in *Distoseptispora* is steadily increasing and currently comprises 35 species, which have been discovered mostly in freshwater and some in terrestrial habitats (Su et al. 2016; Dong et al. 2021; Hyde et al. 2021; Li et al. 2021). Except for the two species, *D.adscendens* and *D.leonensis*, which were found from Hungary and Malaysia, respectively (Shoemaker and White 1985; Mckenzie 1995), 19 of the 33 species has been discovered in Thailand, while the remaining 14 species were introduced from China (Table 2). In China, *Distoseptispora* species are almost exclusively reported in Yunnan Province (Su et al. 2016; Luo et al. 2018; Hyde et al. 2019; Phookamsak et al. 2019; Li et al. 2021). Only three species, *D.martinii*, *D.bambusae* and *D.suoluoensis*, have been discovered from Guizhou Province (Xia et al. 2017; Yang et al. 2018; Sun et al. 2020). In this study, we introduce three new species of *Distoseptispora*, including *D.meilingensis*, *D.yongxiuensis* and *D.yunjushanensis* from Jiangxi Province in subtropical China. We describe the novel species, based on morphological illustrations and phylogenetic analyses. A synopsis of the morphological characters of *Distoseptispora* species is also provided.

## ﻿Materials and methods

### ﻿Samples collection, morphological observation and isolation

Dead bamboo samples from different freshwater habitats in Jiangxi Province, China, were taken to the lab for detection of fungi using a Nikon SMZ-1270 microscope (Nikon Corporation, Japan). Micro-morphological characteristics were observed and captured using a Nikon ECLIPSE Ni-U compound microscope (Nikon Corporation, Japan), equipped with a Nikon DS-Fi3 camera. All measurements were calculated using PhotoRuler Ver. 1.1 software (The Genus Inocybe, Hyogo, Japan) and figures were processed using Adobe Photoshop CS6 Extended version 10.0 software (Adobe Systems, USA). Pure cultures of the fungi were obtained by the single spore isolation method (Chomnunti et al. 2014). The germinating conidia were transferred to potato dextrose agar (PDA) and incubated at 25 °C for two weeks. The fungal cultures were deposited in the Jiangxi Agricultural University Culture Collection (JAUCC) and the holotypic specimens with MycoBank numbers (842065, 842066, 842067) were deposited in the Herbarium of fungi, Jiangxi Agricultural University (HFJAU) .

### ﻿DNA extraction, PCR amplification and sequencing

Fungal genomes were extracted from fresh mycelium using a modified cetyltrimethylammonium bromide (CTAB) method (Doyle and Doyle 1987). Four deoxyribonucleic acid (DNA) barcodes (ITS, LSU, SSU and *Tef*-*1*α) were chosen for polymerase chain reaction (PCR) using the primer pairs ITS1/ITS4 (White et al. 1990), LR0R/LR7 (Hopple and Vilgalys 1999), NS1/NS4 (White et al. 1990) and EF983F/EF2218R (Örstadius et al. 2015), respectively. Amplification reactions were carried out in a volume of 25 μl, containing 12.5 μl 2 × Taq PCR MasterMix (Qingke, Changsha, China), 1 μl each forward and reverse primer (0.2 μM), 1 μl template DNA (circa 50–100 ng) and 9.5 μl ddH_2_O. Amplifications were conducted under the following conditions: 3 min at 98 °C, 35 cycles of 10 s at 98 °C, 10 s of annealing at 55 °C and extension at 72 °C for 10 s, with a final 2-min extension at 72 °C. Sequencing reactions were conducted with the corresponding forward and reverse primers commercially by QingKe Biotechnology Co. (Changsha, China). All sequences were edited with Sequencher v.4.14 (GeneCodes Corporation, USA) and have been deposited in the NCBI GenBank database (Table 1).

**Table 1.** Sequences used in this study.

Taxa	Voucher	LSU	ITS	SSU	*Tef*-*1*α
*Aquapteridosporaaquatica*	MFLUCC 17-2371	NG_075413	NR_172447	—	—
*Aquapteridosporafusiformis*	MFLU 18-1601	MK849798	MK828652	—	MN194056
*Aquapteridosporalignicola*	MFLU 15-1172	KU221018	—	—	—
*Distoseptisporaadscendens*	HKUCC 10820	DQ408561	—	—	—
*Distoseptisporaappendiculata*	MFLUCC 18-0259	MN163023	MN163009	—	MN174866
*Distoseptisporaaquatica*	GZCC 19-0452	MZ227216	MW133908	MW134689	—
*Distoseptisporaaquatica*	MFLUCC 16-0904	MK849794	MK828649	MK828315	—
*Distoseptisporaaquatica*	MFLUCC 18-0646	MK849793	MK828648	—	—
*Distoseptisporaaquatica*	MFLUCC 16-1357	MK849796	MK828650	MK828317	—
*Distoseptisporaaquatica*	S-965	MK849792	MK828647	MK828314	MN194051
*Distoseptisporabambusae*	MFLUCC 20-0091	NG_074430	NR_170068	NG_070348	—
*Distoseptisporabambusae*	MFLU 20-0261	MT232718	MT232713	MT232716	MT232880
*Distoseptisporabambusae*	MFLU 17-1653	MT232717	MT232712	—	—
*Distoseptisporacangshanensis*	MFLUCC 16-0970	MG979761	MG979754	—	MG988419
*Distoseptisporacaricis*	CPC 36498	MN567632	NR_166325	—	—
*Distoseptisporaclematidis*	MFLUCC 17-2145	MT214617	MT310661	MT226728	—
*Distoseptisporaclematidis*	KUN-HKAS 112708	MW879523	MW723056	MW774580	—
*Distoseptisporadehongensis*	KUMCC 18-0090	MK079662	MK085061	—	MK087659
*Distoseptisporaeuseptata*	MFLUCC 20–0154	MW081544	MW081539	—	—
*Distoseptisporaeuseptata*	DLUCC S2024	MW081545	MW081540	—	MW084994
*Distoseptisporafasciculata*	KUMCC 19-0081	NG_075417	NR_172452	—	MW396656
*Distoseptisporafluminicola*	DLUCC 0391	MG979762	MG979755	—	MG988420
*Distoseptisporafluminicola*	DLUCC 0999	MG979763	MG979756	—	MG988421
*Distoseptisporaguttulata*	MFLU 17-0852	MF077554	MF077543	MF077532	MF135651
*Distoseptisporahydei*	MFLUCC 20-0481	MT742830	MT734661	—	—
*Distoseptisporaleonensis*	HKUCC 10822	DQ408566	—	—	—
*Distoseptisporalignicola*	MFLUCC 18-0198	MK849797	MK828651	MK828318	—
*Distoseptisporalongispora*	HFJAU 0705	MH555357	MH555359	MH555431	—
*Distoseptisporamartinii*	CGMCC 318651	KX033566	KU999975	KX033537	—
***Distoseptisporameilingensis***	**JAUCC 4727**	**OK562396**	**OK562390**	**OK562402**	**OK562408**
***Distoseptisporameilingensis***	**JAUCC 4728**	**OK562397**	**OK562391**	**OK562403**	**OK562409**
*Distoseptisporamultiseptata*	MFLUCC 15-0609	KX710140	KX710145	NG_065693	MF135659
*Distoseptisporamultiseptata*	MFLU 17-0856	MF077555	MF077544	MF077533	—
*Distoseptisporaneorostrata*	MFLUCC 18-0376	MN163017	MN163008	—	—
*Distoseptisporaobclavata*	MFLUCC 18-0329	MN163010	MN163012	—	—
*Distoseptisporaobpyriformis*	DLUCC 0867	MG979765	MG979757	—	MG988423
*Distoseptisporapalmarum*	MFLUCC 18-1446	MK079663	MK085062	MK079661	MK087660
*Distoseptisporapalmarum*	MFLU 18-0588	NG_067856	NR_165897	—	MK087660
*Distoseptisporaphangngaensis*	MFLU 17-0855	MF077556	MF077545	MF077534	MF135653
*Distoseptisporaphangngaensis*	MFLUCC 16-0857	—	NR_166230	—	—
*Distoseptisporarayongensis*	MFLUCC 18-0415	NG_073624	NR_171938	NG_073504	—
*Distoseptisporarayongensis*	MFLU 18-1045	MH457137	MH457172	MH457169	—
*Distoseptisporarostrata*	MFLUCC 16-0969	MG979766	MG979758	—	MG988424
*Distoseptisporarostrata*	DLUCC 0885	MG979767	MG979759	—	MG988425
*Distoseptisporarostrata*	MFLU 18-0479	NG_064513	NR_157552	—	—
*Distoseptisporasaprophytica*	MFLUCC 18-1238	NG_075419	NR_172454	—	MW396651
*Distoseptisporasongkhlata*	MFLUCC 18-1234	MW287755	MW286482	—	MW396642
*Distoseptisporasubmersa*	MFLUCC 16-0946	MG979768	MG979760	—	MG988426
*Distoseptisporasuoluoensis*	MFLUCC 17-0224	NG_068552	NR_168764	NG_070113	MF135654
*Distoseptisporasuoluoensis*	MFLU 17-0854	MF077558	MF077547	MF077536	—
*Distoseptisporatectonae*	MFLUCC 15-0981	MW287763	MW286489	—	MW396641
*Distoseptisporatectonae*	MFLUCC 12-0291	KX751713	KX751711	—	KX751710
*Distoseptisporatectonae*	S-2023	MW081543	MW081538	—	—
*Distoseptisporatectonae*	GZ 25	MH555358	MH555361	—	—
*Distoseptisporatectonigena*	MFLUCC 12-0292	KX751714	NR_154018	—	—
*Distoseptisporathailandica*	MFLUCC 16-0270	MH260292	MH275060	MH260334	MH412767
*Distoseptisporathysanolaenae*	KUN-HKAS 112710	MW879524	MW723057	—	—
*Distoseptisporathysanolaenae*	KUN-HKAS 102247	MK064091	MK045851	—	MK086031
*Distoseptisporaxishuangbannaensis*	KUMCC 17-0290	MH260293	MH275061	MH260335	MH412768
***Distoseptisporayongxiuensis***	**JAUCC 4725**	**OK562394**	**OK562388**	**OK562400**	**OK562406**
***Distoseptisporayongxiuensis***	**JAUCC 4726**	**OK562395**	**OK562389**	**OK562401**	**OK562407**
***Distoseptisporayunjushanensis***	**JAUCC 4723**	**OK562398**	**OK562392**	**OK562404**	**OK562410**
***Distoseptisporayunjushanensis***	**JAUCC 4724**	**OK562399**	**OK562393**	**OK562405**	**OK562411**
*Distoseptisporayunnansis*	MFLUCC 20–0153	MW081546	MW081541	—	MW084995

“—”, sequence is unavailable.

### ﻿Data analyses

Reference sequences of 35 *Distoseptispora* species and three *Aquapteridospora* species, based on recent publications (Luo et al. 2019; Hyde et al. 2020; Monkai et al. 2020; Dong et al. 2021, Li et al. 2021) were downloaded from GenBank. Detailed information on fungal strains used in this paper are provided in Table 1.

All obtained sequences were aligned using the online service of MAFFT (Madeira et al. 2019) and refined manually in MEGA v.7.0 (Kumar et al. 2016). Maximum Likelihood (ML) analysis was conducted with RAxML 8.0 using a GTR-GAMMA model of evolution (Stamatakis 2014). Non-parametric bootstrap analysis was implemented using 1,000 replicates to estimate ML bootstrap (BS) values. Bayesian Inference (BI) analysis was carried out with MrBayes v.3.2 under partitioned models (Ronquist et al. 2012). The best-fit models of nucleotide substitutions were selected according to the Akaike Information Criterion (AIC) implemented in jModelTest2.1.1 (Darriba et al. 2012) on XSEDE in the CIPRES web portal (Miller et al. 2010). The models for ITS, LSU, SSU and *Tef*-*1*α datasets used for phylogenetic analysis are GTR+I+G model (-lnL = 4965.1122), GTR+I+G model (-lnL = 2716.7536), TIM2+G (-lnL = 4344.2295) and TrN+I+G (-lnL = 4479.4914), respectively. The datasets were run for 10,000,000 generations, with four chains and trees sampled every 1,000 generations. The first 10% trees were discarded as burn-in. We used three *Aquapteridospora* species as outgroups. The Bayesian consensus tree with posterior probabilities (PP) was visualised with FigTree v.1.4.4 (Rambaut 2018) and was edited in Adobe Illustrator CS6. Our aligned matrices and trees can be obtained from TreeBASE (http://purl.org/phylo/treebase/phylows/study/TB2:S29465).

## ﻿Results

### ﻿Molecular phylogenetic results

**Figure 1.**
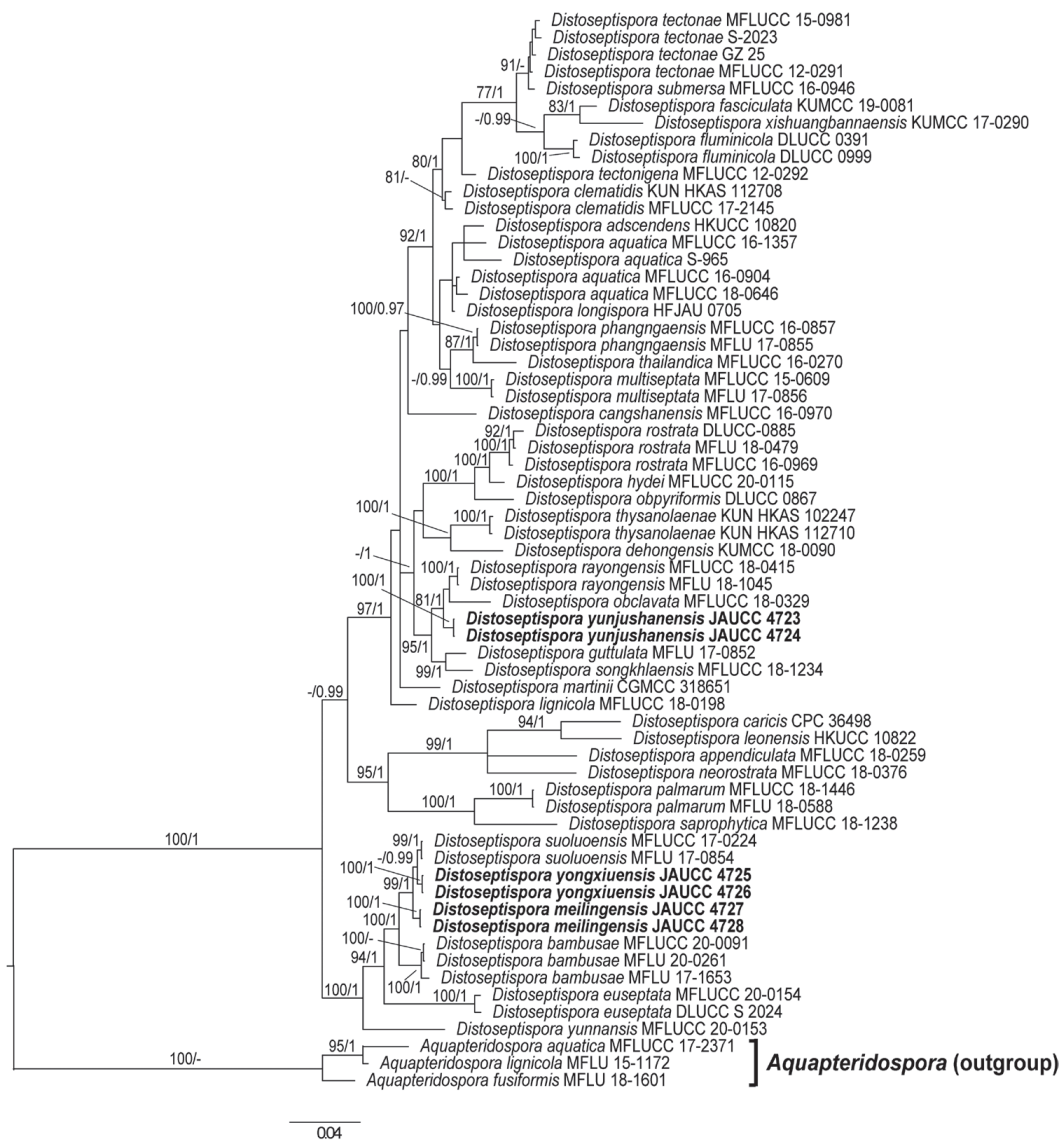
Phylogenetic tree of *Distoseptispora*, inferred from the combined regions (ITS-LSU-SSU-*Tef 1*α) using Bayesian Inference (BI) analysis. The *Aquapteridospora* clade was used as the outgroup. The lineages with new species were shown in bold. PP ≥ 0.95 and BS ≥ 75% were indicated around the branches. The new sequences generated in this study are given in bold.

According to the results of BLAST analysis and sequence alignment, the ITS sequence of *D.meilingensis* has 11 different loci from those of *D.yongxiuensis*, the ITS sequence of which shares 99% similarity (five different loci) with that of *D.suoluoensis*. The ITS sequence of *D.yunjushanensis* is 97% similar (22 different loci) to that of *D.obclavata*. The aligned matrix for the combined analysis, ITS + LSU + SSU + *Tef*-*1*α, had 4015 bp, including ITS 596 bp, LSU 799 bp, SSU 1715 bp and *Tef*-*1*α 905 bp. The topologies of trees generated by ML and BI analyses are highly similar. The Bayesian tree with BS and PP is shown in Fig. 1. All species of *Distoseptispora* form a monophyletic group (BS/PP = 100/1.00). *D.yongxiuensis* groups together with *D.suoluoensis* (BS/PP = 60/0.99). These two species and collections of *D.meilingensis* form a strong-supported clade (BS/PP = 99/1.00), which is strongly linked with sequences of *D.bambusae* (BS/PP = 100/1.00). Collections of *D.yunjushanensis* form a moderate-support clade (BS/PP = 81/1.00) with the lineage consisting of *D.obclavata* and *D.rayongensis*.

### ﻿Taxonomy

#### ﻿ Distoseptispora meilingensis

Taxon classificationFungiDistoseptisporalesDistoseptisporaceae

Z. J. Zhai & D. M. Hu sp. nov.

3AA8B9D3-1DB5-5D94-8DDD-9B3B14BB80A4

 842067

Fig. 2

##### Etymology.

Referring to the collecting site of the Meiling Mountain in Jiangxi Province, China.

##### Holotype.

HFJAU 10009.

##### Description.

Saprobic on culms of bamboo. ***Sexual morph***: Undetermined. ***Asexual morph***: Hyphomycetous. ***Colonies*** effuse, brown to dark brown, hairy. *Mycelium* mostly immersed, composed of pale to dark brown, septate, branched, smooth, hyaline to subhyaline hyphae. ***Conidiophores*** 69–192 × 4–7 μm (x‒ = 120.6 × 5.5 μm, n = 25), macronematous, mononematous, erect, cylindrical, straight or slightly flexuous, 5–12-septate, yellowish-brown or brown, robust at the base. ***Conidiogenous cells*** holoblastic, mono- to polyblastic, integrated, terminal, cylindrical, yellowish-brown or brown. ***Conidia*** 32‒64.5 × (7‒)9‒12.5 μm (x‒ = 43.7 × 9.8 μm, n = 30), acrogenous, solitary, straight or slightly curved, obclavate, 5–7-distoseptate, thick-walled, rounded at the apex, truncate at the base, tapering towards apex, bud scars disjunctors at base, mostly brown when mature.

**Figure 2.**
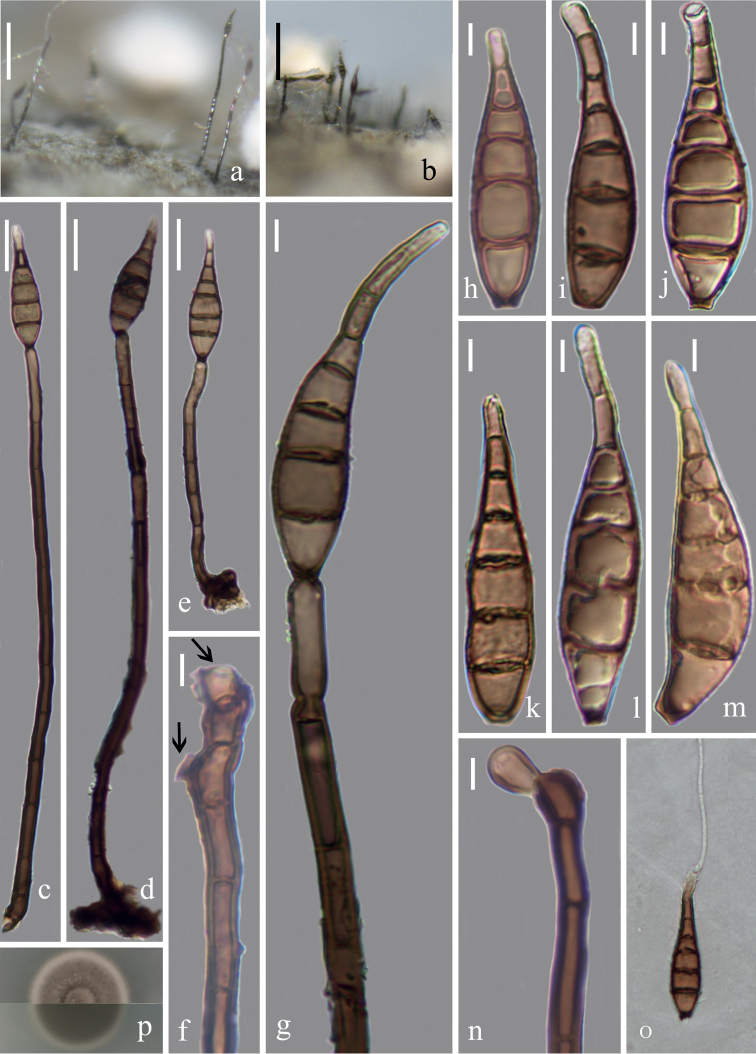
*Distoseptisporameilingensis* (HFJAU10009, holotype) **a, b** colonies on bamboo culms **c–e** conidiophores with conidia **f** conidiogenous cells **g, n** conidiogenous cells with conidia **h–m** conidia **o** germinating conidium **p** culture on PDA from above and reverse. Scale bars: 100 µm (**a, b**), 20 µm (**c–e, o**), 5 µm (**f-n**).

##### Cultural characteristics.

Conidia germinating on PDA within 24 h and germ tubes produced from both ends. Colonies on PDA reaching 17–23 mm diam. at two weeks at 25 °C, in natural light, circular, with dense, light olivaceous mycelium on the surface with entire margin; reverse brown to dark brown.

##### Material examine.

China, Jiangxi Province, Nanchang City, Meiling Mountain, alt. 305 m, near 28.79°N, 115.72°E, on decaying bamboo culms submerged in a freshwater stream, 16 Aug 2021, Z. J. Zhai, SLT-3 (HFJAU10009, ***holotype***), ex-type living culture, JAUCC 4727 = JAUCC 4728.

##### Notes.

*Distoseptisporameilingensis* clusters with the clade including *D.suoluoensis* and *D.yongxiuensis* with high support in the phylogenetic tree (Fig. 1). *Distoseptisporameilingensis* is distinct from *D.suoluoensis* (Yang et al. 2018) and *D.yongxiuensis* by its conidial colour (mostly brown, yellowish-brown to dark olivaceous and yellowish-brown or brown, respectively). Furthermore, *D.meilingensis* has shorter conidia (32–64.5 μm vs. (65–)80–125(–145) μm) than those of *D.suoluoensis* (Yang et al. 2018) and slightly shorter conidiophores (69–192 μm vs. 112–253 μm) than those of *D.yongxiuensis*. *Distoseptisporameilingensis* resembles *D.bambusae* in similar habitats and polyblastic conidiogenous cells (Sun et al. 2020). However, *D.meilingensis* can be distinguished from *D.bambusae* in its longer conidiophores (69–192 μm vs. 40–96 μm), slightly wider (up to 12.5 μm vs. up to 9.5 μm) and brighter (light brown vs. brown) conidia (Sun et al. 2020). A comparison of morphological features of *Distoseptispora* species is provided in Table 2.

**Table 2.** Synopsis of morphological characteristics, habitats, hosts and district compared across *Distoseptispora* species.

Species	Conidiophores (μm)	Conidia (μm)	Conidia septation	Conidia characteristics	Habitat	Host	District	References
***Distoseptisporameilingensis***	**69**‒**192 × 4**‒**7**	**32**‒**64.5 × (7**‒) **9**‒**12.5**	**5**‒**7-distoseptate**	**Obclavate, mostly bright brown when mature**	**Freshwater**	**Dead bamboo culms**	**China, Jiangxi**	**This study**
***D.yongxiuensis***	**112**‒**253 × 4**‒**9**	**46–74** (‒**86)** × **10–13** (‒**16)**	**6**‒**9-euseptate**	**Obclavate or obspathulate, olivaceous to yellowish-brown or brown, guttulate**	**Freshwater**	**Dead bamboo culms**	**China, Jiangxi**	**This study**
***D.yunjushanensis***	**100**‒**175 × 5.5**‒**10**	**39‒67.5** (‒**77) × (7–)9.5–13.5(–16.5)**	**7**‒**13-distoseptate**	**Obpyriform or obclavate, olivaceous when young, dark brown when mature**	**Freshwater**	**Dead bamboo culms**	**China, Jiangxi**	**This study**
*D.adscendens*	28‒46 × 8‒10	(80‒)350‒500 × 15‒18	80-distoseptate	Cylindrical, hemispherical apex, hyaline	Terrestrial	Decaying wood of *Fagussylvatica*	Hungary	Shoemaker and White (1985), Réblová (1999)
*D.appendiculata*	62‒86 × 4.5‒5.5	67‒89 × 10‒16	13‒17-distoseptate	Obpyriform or obclavate, olivaceous or dark brown, with gelatinous sheath around tip	Freshwater	Unidentified submerged wood	Thailand, Khwaeng Phra	Luo et al. (2019)
*D.aquatica*	29‒41 × 7‒9	110‒157 × 13.5‒16.5	15‒28-distoseptate	Obclavate, dark brown with bluish-green to malachite green tinge	Freshwater	Unidentified submerged wood	China, Yunnan	Su et al. (2016)
*D.bambusae*	40‒96 × 4‒5.5	45‒74 × 5.5‒9.5	5‒10-distoseptate	Obclavate, olivaceous or brown	Terrestrial	Dead bamboo culms	China and Thailand	Sun et al. (2020), Monkai et al. (2020)
*D.cangshanensis*	44‒68 × 4‒8	58‒166(‒287) × 10‒14	Multi-distoseptate	Obclavate or lanceolate, rostrate, olivaceous or brown	Freshwater	Unidentified submerged wood	China, Yunnan	Luo et al. (2018)
*D.caricis*	35‒90 × 6‒7	(55‒)65‒85(‒100) × 15‒16(‒17)	5‒10-distoseptate	Obclavate, brown, septa with central pore, basal cell pale brown, with truncate hilum	Terrestrial	Leaves of *Carex* sp.	Thailand, Chiang Mai	Crous et al. (2019)
*D.clematidis*	22‒40 × 4‒10	120‒210 × 12‒20	28‒35-distoseptate	Oblong, obclavate, cylindrical or rostrate, brown with green tinge, bud scars or disjunctors present at the site of attachment	Terrestrial	Dried branches of *Clematissikkimensis*	Thailand, Chiang Rai	Phukhamsakda et al. (2020)
*D.dehongensis*	45‒80 × 4‒5	17‒30 × 7.5‒10	3‒5-distoseptate	Obpyriform to obclavate, broad cylindrical or irregular, olivaceous	Freshwater	Unidentified submerged wood	China, Yunnan	Hyde et al. (2019)
*D.euseptata*	19‒28 × 4‒5	37–54 × 8‒9	4‒7-euseptate	Obpyriform to obclavate, often constricted at septa, olivaceous	Freshwater	Unidentified submerged wood	China, Yunnan	Li et al. (2021)
*D.fasciculata*	12‒16 × 5‒6	46‒200 × 10‒16.5	10‒40-distoseptate	Subcylindrical to obclavate, olivaceous when young, dark brown when mature	Freshwater	Unidentified submerged wood	Thailand, Nakhon Si Thammarat	Dong et al. (2021)
*D.fluminicola*	21‒33 × 5.5‒6.5	125‒250 × 13‒15	17‒34-distoseptate	Oblong, obclavate, cylindrical or rostrate, brown with green tinge	Freshwater	Unidentified submerged wood	China, Yunnan	Su et al. (2016)
*D.guttulata*	55‒90(‒145) × 3.5‒5.5	75‒130(‒165) × 7‒11	11‒14(‒20)-euseptate	Obclavate or lanceolate, rostrate, mid to dark brown or olivaceous	Freshwater	Unidentified submerged wood	Thailand, Prachuap Khiri Khan	Yang et al. (2018)
*D.hydei*	87‒145 × 3‒7	32‒58 × 10‒15	7‒9-distoseptate	Obpyriform to fusiform, olivaceous to brown, with a hyaline, globose, gelatinous sheath around tip	Terrestrial	Dead bamboo culms	Thailand, Phitsanulok	Monkai et al. (2020)
*D.leonensis*	Up to 175 × 6‒7	(38‒)50‒75(‒85) × 11‒15	7‒12-distoseptate	Obclavate, rostrate, brown	Terrestrial	Dead culms of *Freycinetia* sp.	Malaysia	McKenzie (1995)
*D.lignicola*	84‒124 × 4‒5	60‒108 × 7‒9	5‒9-euseptate	Obclavate, curved, brown	Freshwater	Unidentified submerged wood	Thailand, SaiKhu Waterfall	Luo et al. (2019)
*D.longispora*	17‒37 × 6‒10	189‒297 × 16‒23	31‒56-distoseptate	Obclavate, elongated, brown to yellowish-brown	Freshwater	Unidentified submerged wood	China, Yunnan	Song et al. (2020)
*D.martinii*	50‒110 × 3.5‒4.5	15‒20 × 11‒16	Transversal septa	Transversal ellipsoid, oblate or subglobose, muriform, pale brown to brown	Terrestrial	Unidentified dead branches	China, Guizhou	Xia et al. (2017)
*D.multiseptata*	29‒47 × 4‒6	147‒185 × 12‒14	Multi-distoseptate	Obclavate, rostrate, dark olivaceous green	Freshwater	Unidentified submerged wood	Thailand, Prachuap Khiri Khan	Hyde et al. (2016)
*D.neorostrata*	93‒117 × 5.5‒6.5	109‒147 × 13‒15	Multi-distoseptate	Obclavate, rostrate, dark olivaceous to mid or dark brown	Freshwater	Unidentified submerged wood	Thailand, Khwaeng Phra Khanong Nuea	Luo et al. (2019)
*D.obclavata*	117.5‒162.5 × 5‒7	46‒66 × 9‒11	9-11-distoseptate	Obclavate, olivaceous to pale or dark brown, guttulate	Freshwater	Unidentified submerged wood	Thailand, Khwaeng Phra Khanong Nuea	Luo et al. (2019)
*D.obpyriformis*	97‒119 × 5‒7	53‒71 × 12‒16	9‒11-distoseptate	Obpyriform, olivaceous to pale or dark brown, guttulate	Freshwater	Unidentified submerged wood	China, Yunnan	Luo et al. (2018)
*D.palmarum*	90‒165 × 4‒7	35‒180 × 7–11	7‒27-distoseptate	Oblong, obclavate, greenish-black to brown	Terrestrial	Rachis of *Cocosnucifera*	Thailand, Trat	Hyde et al. (2019)
*D.phangngaensis*	18–30(–40) × 4.3‒6.5	165–350 × 14–19	Multi-distoseptate	Elongate, obclavate, rostrate, dark olivaceous to mid or dark brown	Freshwater	Unidentified submerged wood	Thailand, Phang Nga	Yang et al. (2018)
*D.rayongensis*	75–125 × 3.5–5.5	(36–)60–106(–120) × 9–14.5	9‒13-euseptate, rarely 14–15-septate	Obclavate or obspathulate, rostrate, pale brown or pale olivaceous, with percurrent proliferation	Freshwater	Unidentified submerged wood	Thailand, Rayong	Hyde et al. (2020)
*D.rostrata*	82‒126 × 5‒7	115‒155 × 9‒11	(15‒)18‒23-distoseptate	Obclavate or lanceolate, rostrate, olivaceous to pale brown	Freshwater	Unidentified submerged wood	China, Yunnan	Luo et al. (2018)
*D.saprophytica*	50–140 × 3.2–4.2	14.5–30 × 4.5–7.5	2‒6-distoseptate	Subcylindrical to obclavate, olivaceous to brown	Freshwater	Unidentified submerged wood	Thailand, Songkhla	Dong et al. (2021)
*D.songkhlaensis*	70–90 × 4–5.5	44–125 × 9–14.5	9‒16-distoseptate	Obclavate, constricted at septa, olivaceous to brown	Freshwater	Unidentified submerged wood	Thailand, Songkhla	Dong et al. (2021)
*D.submersa*	55‒73 × 7‒9	95‒123 × 15‒19	17‒23(‒28)-distoseptate	Obclavate, brown to dark brown or olivaceous	Freshwater	Unidentified submerged wood	China, Yunnan	Luo et al. (2018)
*D.suoluoensis*	80‒250 × 4.5‒5.8	(65‒)80‒125(‒145) × 8‒13	8‒10-euseptate	Narrowly obclavate or obspathulate, yellowish-brown or dark olivaceous, verrucose, with percurrent proliferation	Freshwater	Unidentified submerged wood	China, Guizhou	Yang et al. (2018)
*D.tectonae*	19.5‒95 × 4.5‒9	45‒270 × 11‒16	10‒40-distoseptate	Obclavate, brown to dark brown or olivaceous	Terrestrial/Freshwater	Dead twig of *Tectonagrandis* (Lamiaceae)	Thailand, Prachuap Khiri Khan	Hyde et al. (2016)
*D.tectonigena*	Up to 110 × 5‒11	(83‒)148‒225(360‒) × (10‒)11‒12(‒13)	20–46-distoseptate	Flexuous, cylindrical-obclavate, elongated, verruculose, dark reddish-brown	Terrestrial	Dead twig of *Tectonagrandis* (Lamiaceae)	Thailand, Chiang Rai	Hyde et al. (2016)
*D.thailandica*	15‒26 × 3‒6	130‒230 × 13.5‒17	35–52-distoseptate	Oblong, obclavate, cylindrical or rostrate, reddish-brown to brown	Terrestrial	Dead leaves of *Pandanus* sp.	Thailand, Prachuap Khiri Khan	Tibpromma et al. (2018)
*D.thysanolaenae*	30‒80 × 3.5‒5.5	21.5‒80 × 6.5‒12.8	8–14-distoseptate	Elongated obclavate, light to dark brown, flat apex, with conspicuous spore attachment loci	Terrestrial	Dead culms of *Thysanolaenamaxima*	China, Yunnan	Phookamsak et al. (2019)
*D.xishuangbannaensis*	12‒17 × 2‒5	160‒305 × 8‒15	Up to 40-distoseptate	Cylindrical-obclavate, green-brown to brown, tapering towards apex	Terrestrial	Dead leaf sheaths of *Pandanusutilis*	China, Yunnan	Tibpromma et al. (2018)
*D.yunnanensis*	131–175 × 6‒7	58‒108 × 8‒10	6‒10-euseptate	Obclavate, rostrate, mid-olivaceous to brown	Freshwater	Unidentified submerged wood	China, Yunnan	Li et al. (2021)

#### ﻿ Distoseptispora yongxiuensis

Taxon classificationFungiDistoseptisporalesDistoseptisporaceae

Z. J. Zhai & D. M. Hu sp. nov.

7BB13B50-3AA3-5003-9F6A-BD3FAD76360A

 842066

Fig. 3

##### Etymology.

With reference to Yongxiu, from where the holotype was collected.

##### Holotype.

HFJAU10007

##### Description.

Saprobic on decaying bamboo culms. ***Sexual morph***: Undetermined. ***Asexual morph***: Hyphomycetous. ***Colonies*** effuse, brown, hairy, glistening, often inconspicuous. ***Mycelium*** partly superficial, partly immersed in the substratum, composed of hyaline to pale brown, septate, branched hyphae. ***Conidiophores*** 112–253 × 4–9 μm (x‒ = 198 × 6.9 μm, n = 15), macronematous, mononematous, solitary or aggregated at the base, cylindrical, straight or slightly flexuous, 8–13-septate, olivaceous to dark brown, sharply curving near the base, paler at the apical part, rounded at the apex. ***Conidiogenous cells*** integrated, terminal, monoblastic, rarely polyblastic, cylindrical, olivaceous to dark brown. ***Conidia*** 46–74(‒86) × 10–13(‒16) μm (x‒ = 65.6 × 12.6 μm, n = 30), acrogenous, solitary, obclavate or obspathulate, straight or flexuous, rostrate, 6–9-euseptate, olivaceous to yellowish-brown or brown, becoming paler or hyaline towards the apex, guttulate, 2.5–4 μm wide at the base and 2.5–5 μm wide at the apex, with a darkened scar at the base.

**Figure 3.**
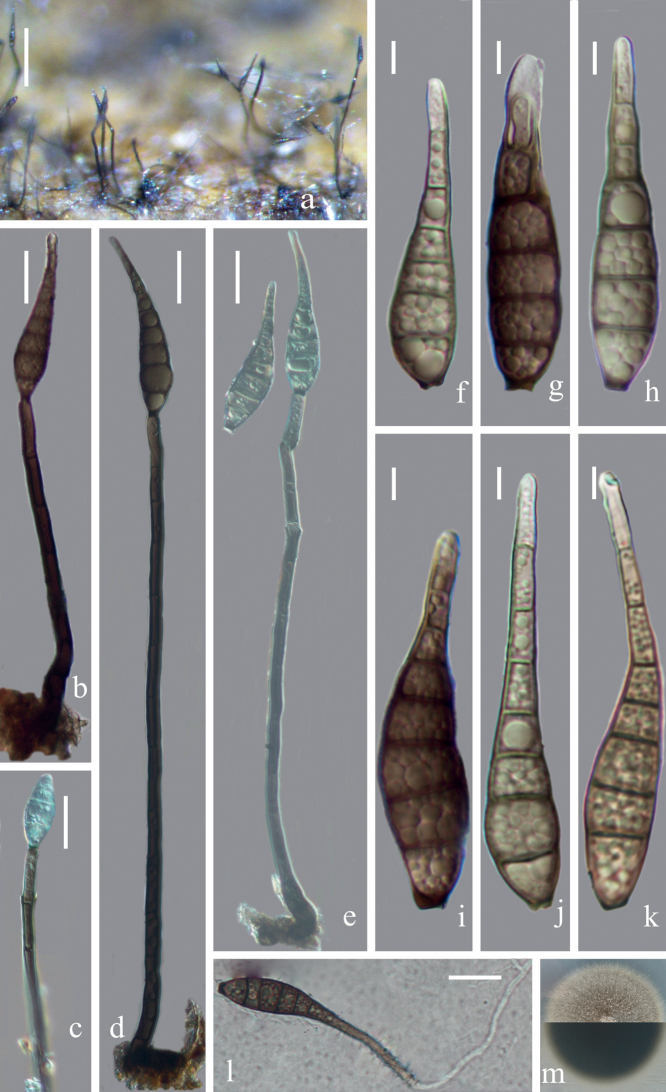
*Distoseptisporayongxiuensis*HFJAU 10007, holotype) **a** Colonies on bamboo culm **b, d** conidiophores with conidia **c** conidiogenous cell bearing conidium **e** conidiogenous cells with young conidia **f-k** conidia **l** germinating conidium **m** culture on PDA from above and reverse. Scale bars: 100 µm (**a**), 20 µm (**b–e, l**), 5 µm (**f–k**).

##### Cultural characteristics.

Conidia germinating on PDA within 24 h and germ tubes produced from both ends. Colonies on PDA reaching 24–32 mm diam. at two weeks at 25 °C, in natural light, circular, with dense, light olivaceous mycelium on the surface with entire margin; reverse dark brown to black.

##### Material examined.

China, Jiangxi Province, Jiujiang City, Yongxiu County, alt. 680.5 m, 29.09°N, 115.62°E, on decaying bamboo culms submerged in a freshwater stream, 28 Apr 2020, Z. J. Zhai and W. W. Li, YJS-70 (HFJAU 10007, ***holotype***), ex-type living culture, JAUCC 4725 = JAUCC 4726.

##### Notes.

In the multi-gene phylogenetic tree (Fig. 1), *D.yongxiuensis* clusters with *D.suoluoensis*. Nonetheless, *D.yongxiuensis* can be distinguished from *D.suoluoensis* by its shorter conidia (46–74(‒86) μm vs. (65–)80–125(–145) μm) and polyblastic conidiogenous cells (Yang et al. 2018). Additionally, *D.suoluoensis* has the percurrent proliferation of conidia, while it was not observed in *D.yongxiuensis*. *Distoseptisporayongxiuensis* is similar with *D.bambusae* (Sun et al. 2020), *D.palmarum* (Hyde et al. 2019) and *D.meilingensis* for the polyblastic conidiogenous cells, but *D.yongxiuensis* has wider conidia than those of *D.bambusae* (10–13(‒16) μm vs. 5.5–9.5 μm) (Sun et al. 2020), shorter conidia than those of *D.palmarum* (46–74(‒86) μm vs. 35–180 μm) (Hyde et al. 2019) and paler (yellowish-brown or brown vs. bright brown) conidia than those of *D.meilingensis*.

#### ﻿ Distoseptispora yunjushanensis

Taxon classificationFungiDistoseptisporalesDistoseptisporaceae

Z. J. Zhai & D. M. Hu sp. nov.

58A74361-CFCB-58AF-868B-8E16DC91B364

 842065

Fig. 4

##### Etymology.

The epithet refers to the collecting site from the Yunjushan Mountain in China.

##### Holotype.

HFJAU10005

##### Description.

Saprobic on decaying bamboo culms submerged in freshwater habitats. ***Sexual morph***: Undetermined. ***Asexual morph***: Hyphomycetous. ***Colonies*** effuse, olivaceous or dark brown, hairy, velvety. ***Mycelium*** mostly immersed, consisting of branched, septate, smooth, subhyaline to pale brown hyphae. ***Conidiophores*** 100–175 μm × 5.5–10 μm (x‒ = 129×7.1 μm, n = 30), single or in groups of 2 or 3, macronematous, mononematous, erect, straight or slightly flexuous, 4–7-septate, unbranched, olivaceous to dark brown, smooth, cylindrical, rounded at the apex. ***Conidiogenous cells*** monoblastic, integrated, terminal, determinate, pale to dark brown, cylindrical. ***Conidia*** 39‒67.5(‒77) μm × (7–)9.5–13.5(–16.5) μm (x‒ = 52 × 12 μm, n = 30), acrogenous, solitary, obpyriform or obclavate, thick-walled, tapering towards the rounded apex, slightly curved, truncate at the base, 7–13-distoseptate, guttulate, smooth-walled, olivaceous, dark brown when mature, sometimes with the percurrent proliferation which forms another conidium from the conidial apex.

**Figure 4.**
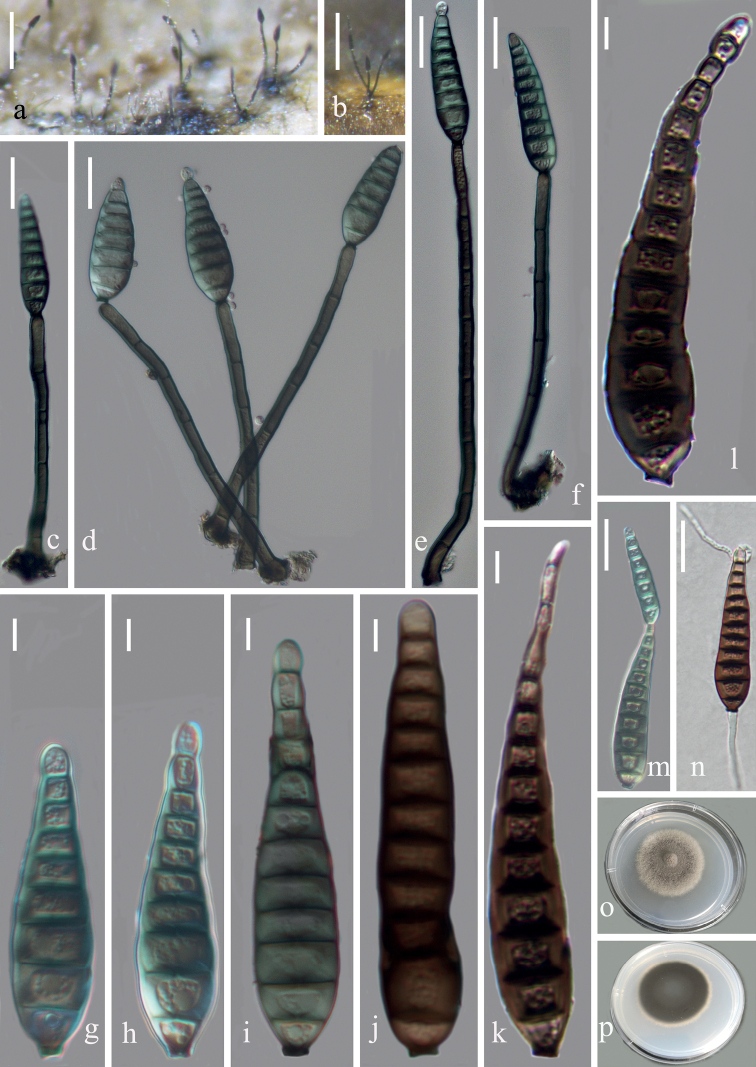
*Distoseptisporayunjushanensis* (HFJAU 10005, holotype) **a, b** colonies on bamboo culms **c–f** conidiophores with conidia **g-i** young conidia **j-l** mature conidia **m** conidium with proliferation **n** germinating conidium **o, p** culture on PDA from above and reverse. Scale bars: 100 µm (**a, b**), 20 µm (**c–f, m, n**), 5 µm (**g–l**).

##### Cultural characteristics.

Conidia germinating on PDA within 24 h and germ tubes produced from both ends. Colonies on PDA reaching 12–18 mm diam. at 14 days at 25 °C, in natural light, with fluffy, dense, thin olivaceous mycelium in the centre, becoming sparse and paler at the entire margin; reverse dark brown, pale brown at the smooth margin.

##### Material examined.

China, Jiangxi Province, Jiujiang City, Yongxiu County, Yunjushan Mountain, alt. 672.5 m, 29.23°N, 115.59°E, on decaying bamboo culms submerged in a freshwater stream, 28 Apr 2020, Z. J. Zhai and W. W. Li, YJS-42 (HFJAU 10005, ***holotype***), ex-type living culture, JAUCC 4723 = JAUCC 4724.

##### Notes.

In the phylogenetic analysis, *D.yunjushanensis* clusters with *D.obclavata* and *D.rayongensis* with moderate support (BS/PP = 81/1.00). However, *D.yunjushanensis* is easily distinguished from *D.obclavata* by its comparatively wider (5.5–10 μm vs. 5–7 μm) conidiophores and conidia ((7–)9.5–13.5(–16.5) μm vs. 9–11 μm) (Luo et al. 2019). Moreover, the percurrent proliferation of conidia was not observed in *D.obclavata* (Luo et al. 2019). *Distoseptisporayunjushanensis* has shorter conidia (39‒67.5(‒77) μm vs. (36–)60–106(–120) μm) and wider conidiophores (5.5–10 μm vs. 3.5–5.5 μm) than those of *D.rayongensis* (Hyde et al. 2020). The morphology of *D.yunjushanensis* is similar to *D.guttulata* and *D.songkhlaensis* in having the obclavate conidia, but differs in having wider (5.5–10 μm vs. 3.5–5.5 μm and 4–5.5 μm) conidiophores, shorter (39–67.5(‒77) μm vs. 75–130(–165) μm and 44–125 μm) and proliferating conidia (Yang et al. 2018; Dong et al. 2021). Additionally, *D.yunjushanensis* can be distinguished from *D.guttulata* by its distoseptate conidia (Yang et al. 2018).

## ﻿Discussion

Previous reports of *Distoseptispora* were mainly concentrated in tropical areas, such as Thailand (Chiang Rai, Phitsanulok, Phang Nga; Luo et al. 2019) and southwest Yunnan, China (Su et al. 2016; Luo et al. 2018). Nonetheless, several new taxa were found sporadically in subtropical China, for example, *Distoseptisporamartinii* (Xia et al. 2017), *D.suoluoensis* (Yang et al. 2018) and *D.bambusae* (Sun et al. 2020) in Guizhou Province and *D.euseptata* and *D.yunnansis* in northwest Yunnan (Li et al. 2021). The ongoing discovery of this taxa from other geographic regions in subtropical China will deepen our understanding of the species in this genus. In this study, we introduced another three new species of *Distoseptispora* from Jiangxi Province of subtropical China. It is interesting to note that all these species in subtropical China, except *D.yunjushanensis* and *D.martinii*, formed a well-supported monophyletic clade in the phylogenetic tree and this clade was at the basal position (Fig. 1). *Distoseptisporayunjushanensis* and *D.martinii* were otherwise phylogenetically placed within other clades (Fig. 1) and, therefore, we suppose that other lineages might also comprise more *Distoseptispora* species distributed in subtropical China. Further discovery of *Distoseptispora* species in more extensive areas in subtropical and other regions of China are needed to be addressed if the phylogenetic position of species reflects their geographical and ecological distribution.

*Distoseptisporaceae* is a holomorphic group of *Sordariomycetes* that are saprobic on decaying wood and plant debris in terrestrial and freshwater habitats (Su et al. 2016). The genus *Distoseptispora* seems not to have specific habitat preferences, as most species were reported from submerged wood in freshwater habitats, while some were introduced from terrestrial habitats (Table 2). So far, only five species of *Distoseptispora* have been found on bamboo, two of them (*Distoseptisporabambusae* and *D.hydei*, Table 2) from terrestrial habitats, the other three (this study) from freshwater. There may be more species in this genus existing on bamboo waiting to be discovered and further studies are needed to clarify if a specific species in *Distoseptispora* is specific to its host.

## Supplementary Material

XML Treatment for Distoseptispora meilingensis

XML Treatment for Distoseptispora yongxiuensis

XML Treatment for Distoseptispora yunjushanensis
